# Genomewide Mutational Diversity in *Escherichia coli* Population Evolving in Prolonged Stationary Phase

**DOI:** 10.1128/mSphere.00059-17

**Published:** 2017-05-24

**Authors:** Savita Chib, Farhan Ali, Aswin Sai Narain Seshasayee

**Affiliations:** National Centre for Biological Sciences, Tata Institute of Fundamental Research, Bengaluru, Karnataka, India; University of Wyoming

**Keywords:** prolonged stationary phase, genome analysis, population genetics

## Abstract

Prolonged stationary phase is an approximation of natural environments presenting a range of stresses. Survival in prolonged stationary phase requires alternative metabolic pathways for survival. This study describes the repertoire of mutations accumulating in starving *Escherichia coli* populations in lysogeny broth. A wide range of mutations accumulates over the course of 1 month in stationary phase. Single nucleotide polymorphisms (SNPs) constitute 64% of all mutations. A majority of these mutations are nonsynonymous and are located at conserved loci. There is an increase in genetic diversity in the evolving populations over time. Computer simulations of evolution in stationary phase suggest that the maximum frequency of mutations observed in our experimental populations cannot be explained by neutral drift. Moreover, there is frequent genetic parallelism across populations, suggesting that these mutations are under positive selection. Finally, functional analysis of mutations suggests that regulatory mutations are frequent targets of selection.

**IMPORTANCE** Prolonged stationary phase in bacteria, contrary to its name, is highly dynamic, with extreme nutrient limitation as a predominant stress. Stationary-phase cultures adapt by rapidly selecting a mutation(s) that confers a growth advantage in stationary phase (GASP). The phenotypic diversity of starving *E. coli* populations has been studied in detail; however, only a few mutations that accumulate in prolonged stationary phase have been described. This study documented the spectrum of mutations appearing in *Escherichia coli* during 28 days of prolonged starvation. The genetic diversity of the population increases over time in stationary phase to an extent that cannot be explained by random, neutral drift. This suggests that prolonged stationary phase offers a great model system to study adaptive evolution by natural selection.

## Abstract (precis)

Prolonged stationary phase in bacteria, contrary to its name, is highly dynamic, with extreme nutrient limitation as a predominant stress. Stationary-phase cultures adapt by rapidly selecting a mutation(s) that confers a growth advantage in stationary phase (GASP). The phenotypic diversity of starving *E. coli* populations has been studied in detail; however, only a few mutations that accumulate in prolonged stationary phase have been described. This study documented the spectrum of mutations appearing in *Escherichia coli* during 28 days of prolonged starvation. The genetic diversity of the population increases over time in stationary phase to an extent that cannot be explained by random, neutral drift. This suggests that prolonged stationary phase offers a great model system to study adaptive evolution by natural selection.

## INTRODUCTION

Microorganisms often face difficult conditions, including nutrient limitation. The model bacterium *Escherichia coli* doubles its population every 30 min during exponential phase in rich laboratory medium. This is in contrast to Savageau’s estimate that in its natural environment—predominantly the lower gut of warm-blooded animals—the average doubling time of *E. coli* might be as long as 40 h (1). Further, in their natural environments, in contrast to standard laboratory media, bacteria are often exposed to a variety of other stresses, including pH variation and oxidative stress (2). In addition, many natural environments are fluctuating in their nutrient content as well as in their presentation of other stresses. Such environments constantly select for genetic variants that are better adapted to the prevailing conditions than their parents were, thus driving evolution. A particular laboratory model for approximating stressful and dynamic environments—characterized by a heterogeneous population of a bacterial species—is prolonged stationary phase (3, 4).

In a typical batch culture of *E. coli* maintained in a controlled environment, bacterial cells divide rapidly and quickly exhaust readily available nutrients (5, 6). Following a brief exponential growth phase, the population makes a transition to the stationary phase wherein resources are diverted to maintenance and survival rather than growth and population expansion (7). After 48 h in stationary phase, the medium is unable to support large populations, resulting in a population crash; the tempo and intensity of the crash vary depending on medium composition as well as the culturing methods adopted (8, 9). The dead cells lyse and supplement the spent medium with potential nutrients. A major component of these potential nutrient resources is amino acids and peptides whose metabolic breakdown results in NH_3_ production that results in an increase in pH of the medium (10). These changes in environment select new genetic variants. This is a continuous process and may extend over a period of several years. This phenomenon is termed growth advantage in stationary phase (GASP), and mutations that confer growth advantages in extended stationary phase are referred to as GASP mutations. The GASP phenomenon was demonstrated in *E. coli* first and has been shown in other bacteria as well (11–13).

The growth advantage conferred by a GASP mutation is typically demonstrated by mixed-culture competition experiments in which a mutant is directly competed against the parent in stationary phase. General trends that have emerged from such GASP studies include the following: (i) a wide spectrum of mutations is selected in a short period of time, resulting in rapid adaptation (11, 14–17); (ii) nutrient limitation is a predominant force of selection as mutants with enhanced ability to scavenge amino acids display GASP phenotypes (16, 18, 19); (iii) there is increased phenotypic diversity, as reflected by colony characteristics, in the population over time (20); (iv) the ever-changing biochemical composition of the population continuously redefines the niche (4); and (v) global regulators of transcription are frequent targets of mutation (11, 14, 16). Despite these studies, the genetic composition of a population of *E. coli* over prolonged stationary phase and its dynamics remain incompletely catalogued and understood.

In this study, we systematically explored the population diversity emerging in *E. coli* populations evolving for 28 days in a lysogeny broth (LB) batch culture incubated without additional nutrient supplementation. Using whole-genome sequencing of population genomic DNA, we catalogue the rise and fall of multiple mutations during prolonged stationary phase, assess the extent to which the observed genetic diversity could be explained by neutral drift, and test for parallelism in the rise of mutations across multiple evolving lines.

## RESULTS

### Evolution in batch culture under prolonged stationary phase.

**FIG 1:**
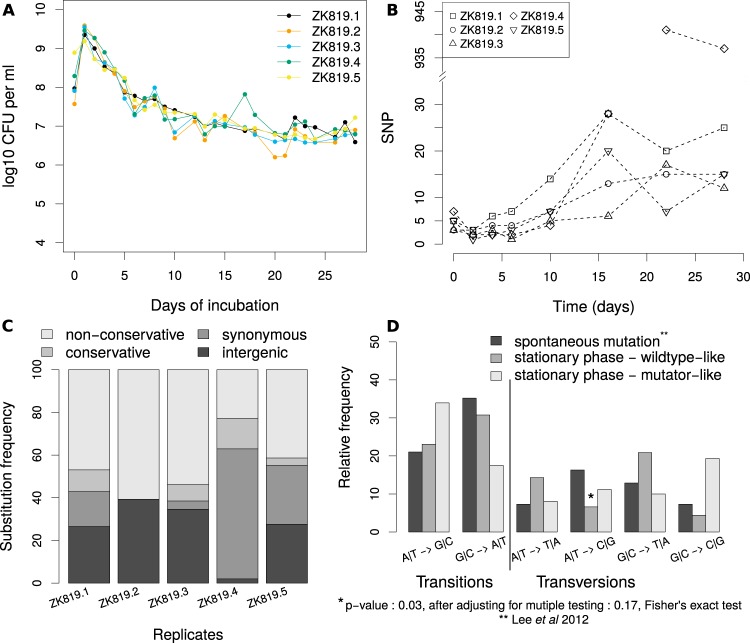
Mutational spectrum analysis in prolonged stationary-phase populations in comparison with mutation accumulation lines. (A) Change in population size over time for five replicate populations. (B) Change in observed number of SNPs over time for five replicate populations. (C) Substitution profiles of five replicates. (D) Comparison of BPS spectra from prolonged stationary phase with that reported for spontaneous mutations in a neutrally evolving population.

We allowed five replicate populations of *E. coli* K-12 ZK819 to stay in stationary phase for 28 days without further supplementation with fresh nutrients. Using the dilution plating method, we found that the drop in viable cell count was gradual with a reduction of approximately 2 orders of magnitude by day 6 (Fig. 1A). Genomic DNA was isolated from a periodically sampled population for next-generation sequencing. ZK819.4 has a mutator-like phenotype (Fig. 1B, diamonds) and is excluded from most of the analysis, except where indicated.

### Status of *rpoS819* during 28 days of evolution.

ZK819 is a W3110 variant originally isolated from an *E. coli* culture evolved in Roberto Kolter’s laboratory under an environment similar to that used in our study (11). ZK819 carries an allelic variant of the stationary-phase sigma factor gene *rpoS*, named *rpoS819*. The *rpoS819* mutation alone is sufficient to confer growth advantage on its bearer under prolonged starvation (11, 18). Since *rpoS819* is a well-known GASP allele that emerges early in prolonged stationary phase, we used this as the parent for our evolution experiment to search for variants that emerge in the background of this previously established GASP mutation.

**FIG 2:**
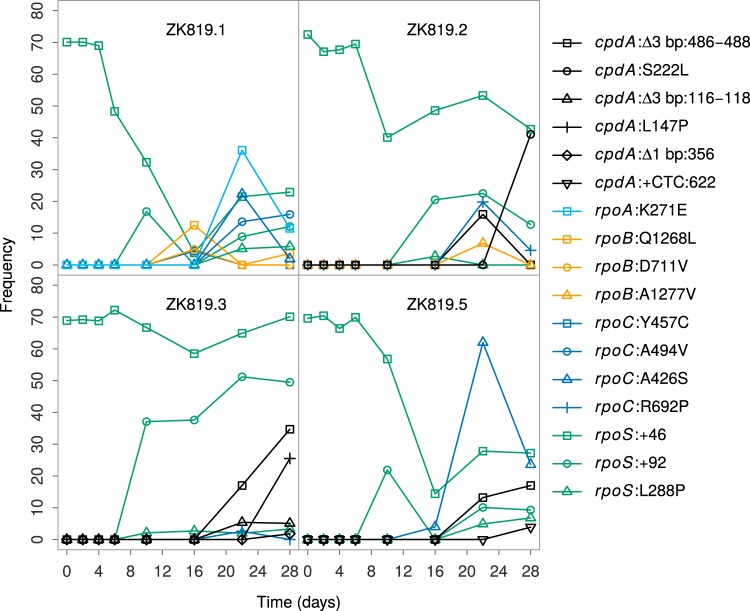
Frequency of ancestral *rpoS819* (stationary sigma factor) and new *rpoS819* allele variant (*rpoS819*_92) fluctuates and exhibits different patterns across replicates (*rpoS*:+46 and *rpoS*:+92). Other genes such as *cpdA* (a cyclic AMP diphosphoesterase) and *rpoC*, *rpoB*, and *rpoA* (RNA polymerase subunits) repeatedly appeared in multiple replicates.

*rpoS819* has a 46-bp duplication at the C-terminal end, resulting in a variant that codes for a longer sigma factor with attenuated activity (11). In addition, we found an A-to-G substitution 21 bp upstream of *rpoS* in all our samples. Sanger sequencing of the DNA upstream of *rpoS* in the genome of the founder strain ZK819 established that it is a preexisting mutation. Genome sequencing data of the population on the first day showed heterogeneity at the* rpoS* locus. The* rpoS819* allele was found in only 70% of the population—as indicated by the proportion of reads supporting the mutation—after 12 h of growth (Fig. 2). The rest is the wild-type (with the preexisting A-to-G substitution upstream of *rpoS*) allele. We identified a third allele of* rpoS* in all replicates from day 10 onward. Sanger sequencing confirmed a 92-bp duplication at the C-terminal end of *rpoS*. Over the course of the experiment, we found this mutant allele—designated *rpoS819_*92—in up to 30% of the population (Fig. 2). Curiously, in some replicates, the combined frequency of *rpoS819* and *rpoS819_*92 is more than 100%. This was explained by Sanger sequencing of *rpoS* from various pure colony isolates, which revealed a fourth allele in the population containing both the 46- and the 92-bp duplications, 4 bp apart from one another. These data suggest that the function of RpoS is constantly tuned and that there is a strong selection favoring it under prolonged stationary phase.

### Spectrum of mutations accumulated over 28 days.

The number of mutations observed by the end of the experiment for four replicates, namely, ZK819.1, ZK819.2, ZK819.3, and ZK819.5, had a mean value of 26 and a range of 22 to 36. Sixty-four percent of these mutations were substitutions (Fig. 1C). Small indels and large deletions/insertions created by insertion elements constituted the remaining set of mutations (Table 1). In the day 22 sample of the ZK819.4 population, we observed a nearly 40-fold increase in substitution mutations. We therefore refer to ZK819.4 as mutator-like.

**TABLE 1:** Mutational spectra at 28 days of five replicate populations in prolonged stationary phase

Mutation type	No. of mutations in population:				
	ZK819.1	ZK819.2	ZK819.3	ZK819.4	ZK819.5
SNP	49	28	26	1,102	29
Insertion	2	2	2	8	3
Deletion	7	11	7	19	7
Large deletion	8	3	4	4	4
Total	66	44	39	1,133	43
Intergenic	22	14	13	29	13
Coding	44	30	26	1,104	30

Spontaneous mutations in the absence of selection are expected to leave a characteristic spectrum of base pair substitutions (BPS), deviations from which may indicate the presence of mutagens or the absence of specific mismatch repair pathways or even the presence of selection. A mutation accumulation (MA) protocol is most appropriate for scanning the unbiased pool of spontaneous mutations, as it minimizes the influence of selection by its repetitive single-individual bottleneck strategy. We compared our data on BPS to those from a previously published MA study on wild-type *E. coli* (21). The data from four replicates excluding ZK819.4 were pooled for this comparison.

Spontaneous mutations are known to exhibit a G|C→A|T bias. The fraction of G|C→A|T substitutions among transitions was higher than that of A|T→G|C substitutions in all four populations except the mutator-like ZK819.4 (Fig. 1D). The occurrence of these substitutions did not significantly differ from those reported from the MA experiments for the wild type, except for underrepresentation of A|T→C|G (Fisher exact test, *P* value of 0.029, significance threshold of 0.05); this significance disappears upon correcting for multiple comparisons. On the other hand, the fractions of both types of transitions and that of G|C→C|G transversions in the mutator line ZK819.4 were significantly different from those in the wild-type MA line (Fig. 1D).

The MA study had reported a shift in BPS from G|C→A|T to A|T→G|C in a *mutL* strain, which lacks a component of the mismatch repair pathway. We observed the same shift in ZK819.4; in addition, we also observed a dramatic increase in G|C→C|G substitutions which was not observed in the MA experiment for *mutL* (Fig. 1D). We did not find any mutation in the methyl-directed mismatch repair system in ZK819.4; however we found that the ZK819.4 population carried a nonsynonymous mutation in *uvrB* (F497Y) at a frequency of ~7% on day 22.

Overall, the BPS spectrum in prolonged stationary-phase populations excluding ZK819.4 was only marginally different from the BPS spectrum resulting from spontaneous mutations in wild-type *E. coli* (Pearson’s chi-square contingency table test, *P* value of 0.01 to 0.05, 2,000 Monte Carlo simulations). The BPS spectrum of the mutator population, however, was significantly different from those of other populations as well as from those of the MA lines from the wild-type and the *mutL E. coli* (Pearson’s chi-square contingency table test, *P* value of <2 × 10^−3^, 2,000 Monte Carlo simulations). In the MA study, the fraction of nonsynonymous substitutions was 0.69 and 0.66 in the wild-type and the *mutL* lines, respectively, which is close to the *a priori* random expectation. In ZK819.4, at the last two time points where an excessive number of substitutions was observed, the fraction of nonsynonymous substitutions was around 0.36. For the corresponding time points, this fraction across the other replicates was on average 0.88 and 0.66, respectively. The variability in these numbers across time points does not allow us to develop a firm interpretation of these results.

### Functional conservation at mutated residues across orthologs.

Functional constraints on an amino acid residue in a protein are expected to keep the residue conserved across its evolutionarily close orthologs. The residue itself may, however, be allowed to be replaced as long as the physicochemical properties at that residue position in the protein are not altered.

Therefore, conservation of a certain residue type at any given position across a majority of a protein’s orthologs would indicate that a mutation that alters the properties of that residue might affect the activity of the protein.

**FIG 3:**
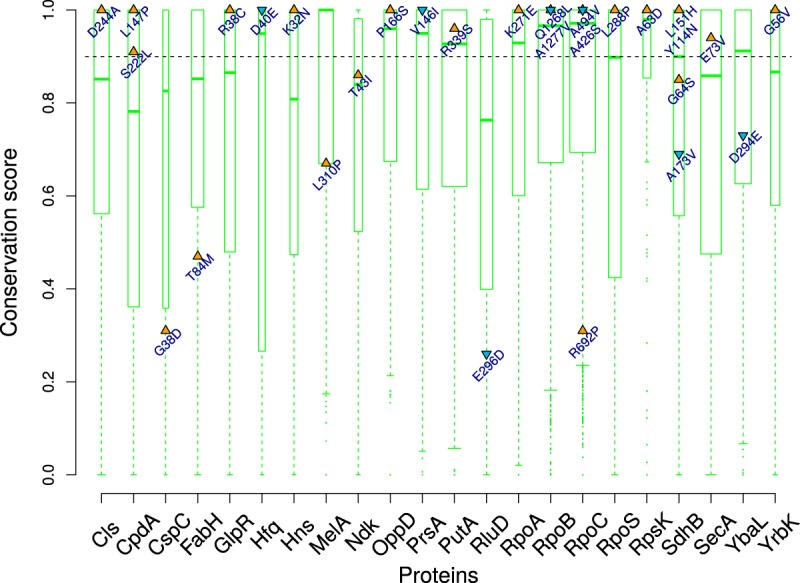
Highly conserved amino acid residues were mutated in prolonged stationary phase. The conservation score was calculated for each protein using Shannon’s entropy (see Materials and Methods). The width of boxes is indicative of protein size. The dashed line marks the average median score for these proteins. Triangles mark the score for mutated residues in each protein, and orange indicates a nonconservative amino acid change while blue indicates a conservative one.

With this reasoning, we scored the level of functional conservation, across gammaproteobacterial genomes, at residue positions affected by nonsynonymous mutations. We restricted this analysis to mutations appearing at above a 5% frequency in the population. We searched for orthologs of proteins carrying these nonsynonymous mutations across 246 gammaproteobacteria. We defined a conservation score, ranging from 0 to 1, for each residue (see Materials and Methods). We calculated conservation scores for all the residues of a protein by normalizing their Shannon entropy values between 0 and 1 such that a score of 1 would represent the most conserved residue in the protein and vice versa. For the 22 proteins considered here, the average median score was around 0.9. Twenty out of 29 mutated residues scored above 0.9, 17 of which scored 1 (Fig. 3). These observations suggest that most of the mutated residues occupy well-conserved positions in respective proteins.

We noted that 22 of these nonsynonymous mutations altered the physicochemical class of the residue concerned. Since a majority of these mutations were at conserved positions, these can be expected to be of functional consequence for their respective proteins.

### Genetic diversity in prolonged-stationary-phase populations.

**FIG 4:**
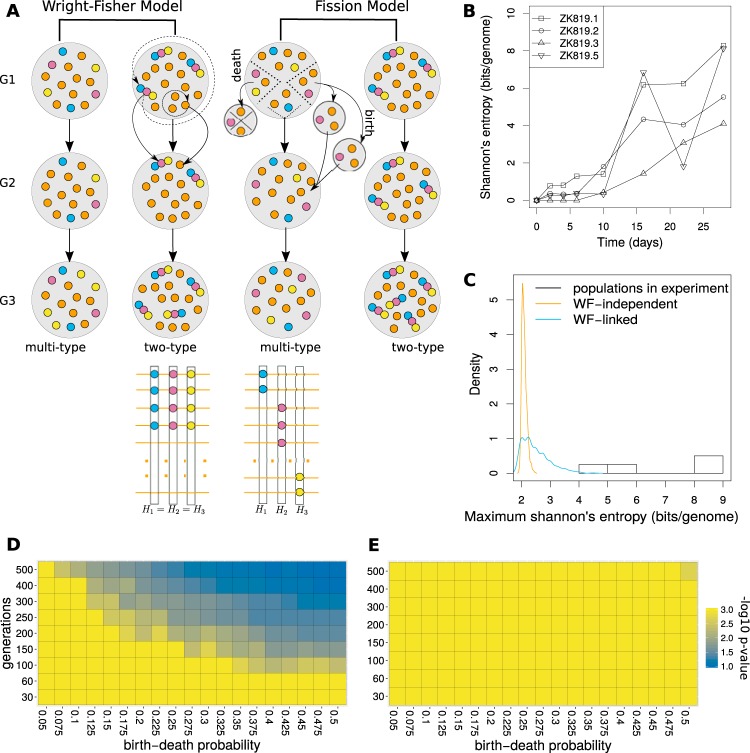
Simulation of neutral evolution in prolonged stationary phase. (A) Schematic for Wright-Fisher and fission models used for neutral evolution simulation, illustrated by running a simulation with a population of 15 individuals for 3 generations and calculating Shannon’s entropy at each mutated position in the genome. Orange circles indicate the ancestral allele; other colors indicate mutant alleles. Circles stuck together indicate all mutant alleles to be present on the same genome. (B) Change in Shannon’s entropy of the whole genome (calculated from frequency of observed substitutions in the population) over time for 4 replicates. (C) Distribution of maximum Shannon’s entropy over 20 days for 1,000 simulations of Wright-Fisher model in comparison with the values observed in 4 replicates. (D and E) Parameter scan for birth-death probability and number of generations in 20 days under fission model. (D) Two type. (E) Multitype.

We measured the genetic diversity of the population at each time point of our experiment by measuring Shannon’s entropy of the genome (22). In each replicate, we observed an overall increase in genetic diversity with time, but the rate of change at each time point varied across replicates, with some time points even showing a brief decline in the measured genetic diversity (Fig. 4B).

We tested through simulations the null hypothesis that the observed level of genetic diversity is attainable under neutral evolution with the same number of substitutions and initial mutant frequencies as those for a prolonged-stationary-phase population. We simulated neutral evolution assuming a constant population size, which was appropriate for our prolonged-stationary-phase population from day 10 onward. In these simulations, new mutations did not occur *de novo*, and all mutations were considered to have been present at a frequency of 1% at the initial time point. We considered ZK819.1 the representative case for this analysis, because it has the highest number of substitutions at the last time point and the entropy at the starting time point can be explained by considering all its 25 substitutions at a frequency no greater than 1%. The genetic diversity of ZK819.1 reaches its maximum of 8 bits/genome by the last time point, corresponding to an average mutant frequency of 5% per site. We note that our measure of genetic diversity represents an upper estimate of true diversity in the genome because all substitutions are considered completely independent, i.e., the knowledge of a substitution at one position does not provide any information about the presence or absence or the nature of a substitution at another position. The value of this measure would remain the same even under the extreme condition where all substitutions constitute a single haplotype. Therefore, in our neutral evolution simulations, we considered both the extreme cases: one where we have a single haplotype with 25 substitutions and another where each substitution is free to be part of any haplotype (Fig. 4A). For the former case, we needed to simulate change only in the frequency of a single allele initially present in 1% of the population. For the latter case, we needed to observe the change in entropy in a genome of 25 independent substitutions, where initially all the substitutions were at 1% frequency in the population. The probability of attaining the observed value of Shannon’s entropy is expected to be higher in the case of a single mutant haplotype. We note here that the chance that a single mutant haplotype scenario would be applicable to our system is low, in light of the fact that the frequency profiles of many pairs of mutations are uncorrelated.

We considered a relatively smaller population of bacteria for simulations (10^4^, approximately 0.1% of our population size estimates from colony-counting experiments). Frequency changes become less prominent in a larger population, and so, the probability of mutations attaining a high frequency in a larger population under neutral evolution would be even lower than the simulated *P* values for a population of 10^4^ bacteria.

Under the Wright-Fisher (WF) model for neutral evolution, both cases failed to attain the observed genetic diversity (*P* value of <10^−3^; 1,000 iterations) (Fig. 4C). Even with a 4-fold increase in the number of WF generations, the *P* value for a single mutant haplotype scenario was 0.003. We then turned to the fission model, which is considered a more realistic drift model for bacterial populations. Since the number of generations covered by our evolution experiment and the birth-death rate probability in prolonged-stationary-phase populations are unknown, we performed simulations with various values of these two parameters (Fig. 4D and E). We observed an increase in the probability of finding the observed genetic diversity with increasing number of generations and birth-death probability. When all substitutions were considered independent, the *P* value for the observed genetic diversity with a birth-death probability of 0.5 and 500 generations was 2 × 10^−3^ (1,000 iterations). For all other parameter combinations, it fell below 10^−3^. Under the extreme, unlikely case where all substitutions constitute a single haplotype, the probability of observing a genetic diversity as high as that in the prolonged-stationary-phase populations did not remain significantly low for more than 150 generations for higher values of birth-death probability.

To get an upper estimate of number of generations in prolonged stationary phase, we considered the case of a mutation that appeared on day 16 at 100% frequency while it was not observed on day 10. This mutation either could be present on day 10 but below the level of detection or could have appeared at any time between day 10 and day 16. If we consider this mutation to have originated as late as day 13 and to have increased in numbers from 1 to 10^7^ in 3 days, our estimate of number of generations under logarithmic growth assumptions would be 1/3 × log_2_ 10^7^ = 8 generations/day, which sums to no more than 160 generations in 20 days of prolonged stationary phase. Given this, the probability that neutral drift can explain the observed level of genetic diversity is low. Therefore, we can infer that the genetic diversity of prolonged-stationary-phase populations is a signature of evolution under natural selection.

### Functional bias in frequencies of mutations observed in prolonged stationary phase.

Since the mutations were randomly sampled, we asked if we observed any bias in relative proportions of functional classes represented in the pool of mutations. If we observed a bias toward a functional category, it would mean that among the observed mutations, a larger-than-expected proportion is sustained in that category in prolonged stationary phase.

Using the MultiFun classification scheme, we classified all coding region mutations (*n =* 93, of which 10 were synonymous) into 6 functional categories based on the functions of their carrier genes. To have sufficient power in the statistical test, we pooled the mutations from the 4 nonmutator replicate populations and set the experiment-wide significance threshold to 0.01.

**FIG 5:**
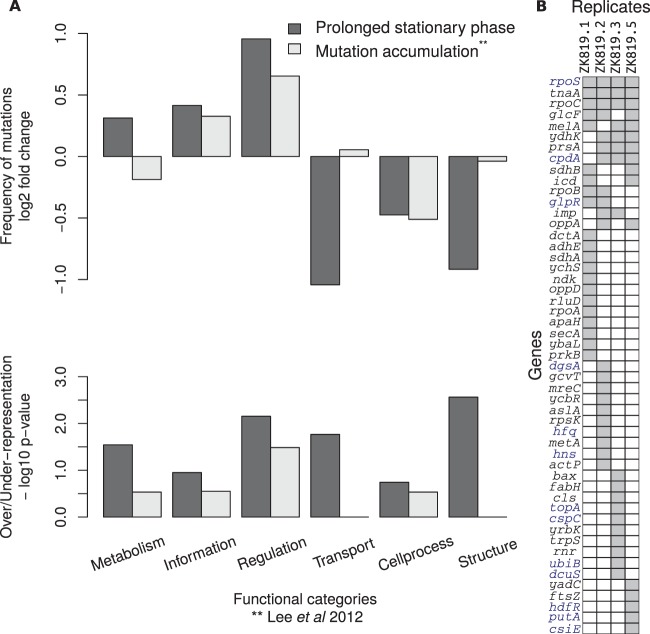
Functional bias among mutations observed in prolonged stationary phase. (A) The top plot shows deviation (log_2_ fold change) from the number of mutations expected in each functional category based on its relative size (total nucleotides) in the genome. The bottom plot shows corresponding −log_10_ *P* values (adjusted for multiple testing with Holm-Bonferroni method) for over- and underrepresentation. The “Regulation” category appears to be overrepresented in prolonged stationary phase; no such significant bias was observed for spontaneous mutations under the neutral mutation accumulation experiment of Lee et al. (21). (B) Binary matrix for genes mutated in four replicates; gray marks their presence. The genes highlighted in blue type have regulatory functions.

We found that the category “Regulation” was overrepresented among the observed mutations with a *P* value of 0.007, whereas “Structure” was observed to be significantly underrepresented (Fig. 5A). Only one out of the 22 mutations in genes categorized under “Regulation” was synonymous. All mutations observed in regulatory genes have been listed in Table 2.

**TABLE 2:** Some mutations of interest with relevant details and maximum frequency over 28 days in each replicate^a^

Gene	Position	Mutation	Annotation	Maximum frequency (%) in replicate:			
				1	2	3	5
*cpdA*	3175000	Δ3 bp	Coding (488–490/828 nt^b^)	0	15.9	34.7	17
*cpdA*	3174825	G→A	S222L (TCG→TTG)	0	41.1	0	0
*cpdA*	3175372	Δ3 bp	Coding (116–118/828 nt)	0	0	5.4	0
*cpdA*	3175050	A→G	L147P (CTG→CCG)	0	0	25.5	0
*cpdA*	3175134	Δ1 bp	Coding (356/828 nt)	0	0	1.8	0
*cpdA*	3174868	+CTC	Coding (622/828 nt)	0	0	0	3.9
*csiE*	2664350	T→C	L87S (TTA→TCA)	0	0	0	3.1
*cspC*	1909037	C→T	G38D (GGT→GAT)	0	0	6.1	0
*dcuS*	4355098	G→T	L415M (CTG→ATG)	0	0	1.9	0
*dgsA*	1669599	Δ1 bp	Coding (680/1,221 nt)	0	100	0	0
*glpR*	4079921	C→T	R38C (CGC→TGC)	9.9	0	0	0
*glpR*	4080435	C→A	S209* (TCG→TAG)	0	2.7	0	0
*hdfR*	3689274	G→A	W187* (TGG→TGA)	0	0	0	1.4
*hfq*	4405087	T→A	D40E (GAT→GAA)	0	26.7	0	0
*hns*	1294404	T→A	K32N (AAA→AAT)	0	6.8	0	0
*putA*	1078290	G→T	R339S (CGC→AGC)	0	0	0	9.8
*putA*	1078496	G→T	A270D (GCC→GAC)	0	0	0	10.8
*rpoS*	2865274	+46 bp	Coding (817/876 nt)	70.1	72.5	72.2	70.4
*rpoS*	2865278	+92 bp	Coding (813/876 nt)	16.8	22.5	51.2	21.9
*rpoS*	2865228	A→G	L288P (CTG→CCG)	5.8	2.7	3.3	6.8
*topA*	1335341	G→T	W860C (TGG→TGT)	0	0	2.2	0
*ubiB*	3615064	C→A	L464L (CTG→CTT)	0	0	3.5	0
*rpoA*	4200197	A→G	K271E (AAA→GAA)	36.1	0	0	0
*rpoB*	3451634	T→A	Q1268L (CAG→CTG)	12.5	0	0	0
*rpoB*	3453305	T→A	D711V (GAC→GTC)	4.9	0	0	0
*rpoB*	3451607	G→A	A1277V (GCG→GTG)	0	6.8	0	0
*rpoC*	3449962	T→C	Y457C (TAT→TGT)	4.4	0	0	0
*rpoC*	3449851	G→A	A494V (GCG→GTG)	15.9	0	0	0
*rpoC*	3450056	C→A	A426S (GCA→TCA)	22.3	0	0	62
*rpoC*	3449257	C→G	R692P (CGT→CCT)	0	19.8	2.6	0
*sdhB*	758440	C→A	F110L (TTC→TTA)	100	0	0	0
*sdhB*	758300	G→A	G64S (GGT→AGT)	1.7	0	0	9.9
*sdhB*	758562	T→A	L151H (CTC→CAC)	16	0	0	0
*sdhB*	758579	A→T	T157S (ACC→TCC)	1.8	0	0	0
*sdhB*	758631	G→T	G174V (GGC→GTC)	1.2	0	0	0
*sdhB*	758568	C→A	A153E (GCA→GAA)	2.5	0	0	0
*sdhB*	758628	C→T	A173V (GCA→GTA)	10.8	0	0	0
*sdhB*	758450	T→A	Y114N (TAT→AAT)	0	0	0	100

a These mutations include mutations in genes classified as regulatory in MultiFun and in genes involved in RNA polymerization.

b nt, nucleotides.

To test if spontaneous mutations have any inherent bias toward any functional category, we performed the same analysis on data on spontaneous mutations in wild-type *E. coli* from a mutation accumulation study (21). We observed that in these MA data, the class “Regulation” was only marginally overrepresented (*P* value of 0.03).

Five of the 50 genes observed to be mutated in prolonged-stationary-phase populations (*glcF*, *hdfR*, *csiE*, *bax*, and *ychS*) lacked a MultiFun annotation. We could include the first four of these in the analysis as the required functional information was available in other databases and the literature. A *ychS* allele variant with 3 point mutations, of which 2 are nonsynonymous, was identified in ZK819.1 at the day 16 time point at 100% frequency. *ychS* encodes a 91-amino-acid protein for which no additional information is available in *E. coli* databases.

### Genetic parallelism during prolonged stationary phase.

Among the mutations identified are a set of genes that appeared in multiple replicates during roughly the same period of the evolution experiment (Fig. 5B). We have mentioned the case of *rpoS819_*92 above (Fig. 2 and 5B). We observed that the components of the RNA polymerase are frequently targeted under prolonged starvation. *rpoC* alleles appeared in four evolving populations. Replicate 2 has mutations in* rpoB* and *rpoC*. Replicate 1 has mutant variants of *rpoC*, *rpoB*, and *rpoA*. *rpoA* has an A-to-G point mutation (K271E) that has been previously characterized and shown to be crucial for RpoA α-C-terminal domain (α-CTD) interaction with cyclic AMP (cAMP) receptor protein (CRP) at some of the CRP-dependent promoters (23, 24). These mutants rose in frequency for a period and were in decline by the end of the evolution experiment (Fig. 2).

Another case of parallel evolution is mutations in the *cpdA* gene. CpdA is a cAMP phosphodiesterase that hydrolyzes cAMP (25). We identified multiple alleles of *cpdA* in four out of five replicates, except ZK819.1. We successfully isolated two alleles of *cpdA* from ZK819.3, a T-to-C point mutation that changes leucine to proline in an alpha helix, and a 3-nucleotide deletion (position 486 to 488) that knocks off a leucine residue which immediately precedes the stretch of metal binding histidine residues. Both these *cpdA* alleles were present in the *rpoS819_*92 background. These two *cpdA* alleles increase in frequency over time. The 3-nucleotide deletion (position 486 to 488) independently appeared in four replicates. These two mutations are likely to negatively affect the function of CpdA. Another locus of interest is the *lptD* (*imp*) gene. LptD is an essential outer membrane protein which along with LptE functions in the assembly of lipopolysaccharides at the surface of the outer membrane (26). A 21-nucleotide deletion at the N-terminal region of the* lptD* gene independently appeared in ZK819.2 and ZK819.3. The deletion of 21 nucleotides results in the deletion of 7 amino acids. Like *cpdA* alleles, *lptD* alleles appeared during the later stages of the experiment.

## DISCUSSION

Long-term stationary phase is a dynamic environment characterized by a multitude of stresses with nutrient limitation as a predominant stress. Previous studies of bacterial evolution in long-term stationary phase have shown the continuous emergence of variants which outcompete their parents (3, 11). A few such variants have been genetically identified (11, 14–16). However, much of the variability has been described phenotypically, for example, by examination of colony morphotypes (20). These have indicated that *E. coli* populations in long-term stationary phase are neither homogeneous nor static. In the present study, we have used population genome sequencing to reinforce this at a genetic level.

We show that genetic diversity increases over time in long-term stationary-phase populations. It has been suggested that stress could accelerate the generation of genetic diversity by inducing the expression of error-prone DNA polymerases and a reduction in certain repair activities or by selecting for genetic variants that result in mutator phenotypes (27–37). In our study, one out of the five populations displayed a strong mutator phenotype. The contribution, if any, of stress-induced mutagenesis to the patterns that we observe is an open question. The expression of various error-prone DNA polymerases is dependent on RpoS (29, 31). Attenuated status of RpoS in our long-term stationary-phase cells might dampen the argument in favor of selective induction of these DNA polymerases and their role in generating genetic diversity. Moreover, we have shown that the base pair substitution rates in nonmutator lines in prolonged stationary phase are not significantly different from the underlying mutational spectrum of wild-type *Escherichia coli* by making comparisons with data from a mutation accumulation study, which reveals the spectrum of mutations that can occur given neutral drift (minimal selection) under the growth conditions tested (21). This observation suggests that the substitutions in prolonged stationary phase are generated by mutational processes that are similar to those tested in the MA study. This comparison of prolonged stationary phase with MA was not aimed at drawing any similarities between these two experimental systems. At this time, it is unclear how the equivalent of an MA experiment that samples conditions closer to those observed in prolonged stationary phase can be designed.

Can the level of genetic diversity observed in our study be explained by neutral drift, or was it achieved under selection? The role of neutral evolution versus natural selection in the evolution of genetic diversity is a subject of great debate, especially in view of arguments from Michael Lynch that even complex regulatory network architectures can evolve purely by drift (38). A recent study of mutations in an exponential-phase laboratory evolution experiment had used parameters such as the number of nonsynonymous versus synonymous variations to indicate that many of the mutations observed in the study are under selection (39). The relatively smaller numbers of mutations in our nonmutator lines, and the variability in such a parameter across lines, did not permit a similar analysis. However, a comparison of the genetic diversity that we had observed against what would be predicted by two different models of evolution under pure neutral drift reinforced the view that evolution of genetic diversity in long-term stationary phase is driven by selection. Further, many nonsynonymous mutations that we see will result in a switch in the physiochemical characteristics of residues that are conserved to a high degree across bacteria. The prevalence of the repeated occurrence of a large fraction of mutations in certain genes across multiple lines might also be evidence for selection favoring these variants. A recent study has shown that populations evolve rapidly under complex environmental conditions involving a variety of stresses wherein genetic parallelism appears to be a prevalent feature (17). We observed a strong enrichment of mutations in regulatory genes, which was not the case for a pool of spontaneous mutations under minimal selection (MA data). Moreover, several of these regulators were independently mutated in more than one replicate population. This may suggest strong genetic parallelism and selection favoring mutations with pleiotropic effects. This includes further duplications within* rpoS*. One or more RNA polymerase subunit genes were frequently mutated in four of the five replicates, suggesting global changes in transcription.

A strong case of this genetic parallelism is reflected at the *cpdA* locus. The appearance of various *cpdA* alleles with unique mutations in four of the five populations suggests that there is a strong selection for *cpdA* genetic variants. cAMP-CRP and RpoS control the two largest regulons in *E. coli* by controlling alternate carbon utilization and general stress response during stationary phase (40–43). However, the interaction between these regulators is unclear. Frequent mutations in *rpoS* and the *cpdA* locus in our study may suggest remodulation of regulatory interactions between these two regulatory networks, generating adaptation under prolonged stationary phase. Experiments to evaluate the fitness effect of *rpoS* and *cpdA* mutations and the underlying molecular mechanisms are under way in our laboratory. Evolution by variation in regulatory networks is rapidly gaining ground through various examples in natural populations as well as in laboratory evolution, and the present study provides yet another example in this field.

## MATERIALS AND METHODS

### Evolution experiment.

Five biological replicate cultures of *E. coli* K-12 strain ZK819—a derivative of W3110—were seeded at a 1:1,000 dilution in lysogeny broth (LB), from overnight-grown cultures. The starting culture volume was 200 ml in a 500-ml-capacity flask. The incubator shaker was set at 37°C and 200 rpm. To compensate for evaporation, 1 ml of sterile water was added every alternate day. Addition of water did not compensate for the culture volumes withdrawn for making glycerol stock, plate cell count, and genomic DNA isolation. Population size for each replicate was tracked by serial dilution plating of 0.1 ml of the culture every 24 h during the experiment. One milliliter of culture was drawn from the evolving population each day, and duplicate glycerol stocks—for storage at −80°C—were made. One milliliter of sample was drawn from each evolving population during the first-week time points to extract genomic DNA, and 5 ml was drawn for the rest of the 3-week time points for whole-genome resequencing. Two hundred nanograms of genomic DNA was sheared, of which 50 ng was used for library preparation.

### Sequencing and data analysis.

Genomic DNA was isolated from a sample of the evolving population at days 0, 2, 4, 6, 10, 16, 22, and 28. The isolation was performed using the GenElute bacterial genomic DNA kit (Sigma-Aldrich), according to the manufacturer’s protocol. Sequencing libraries were prepared using Illumina’s TruSeq Nano DNA LT library preparation kit. Whole-genome sequencing was performed at the Centre for Cellular and Molecular Platforms (C-CAMP) on the Illumina HiSeq1000 platform according to the manufacturer’s instructions. The mean number of 101-base-long reads mapped to the reference genome for 40 sequenced samples was 13.45 million, resulting in a mean coverage of 292×.

The reads were mapped to the reference *E. coli* genome W3110 (GenBank accession no. NC_007779.1), and putative single nucleotide polymorphisms (SNPs), small indels, and structural variants were called using the breseq pipeline, which uses Bowtie for sequence alignment (44, 45). Variant calling for the founder population was done to identify preexisting mutations. breseq was run with its default parameters (using the -p option to identify mutations covered by only a subset of reads), and only those mutations predicted with high confidence (under the heading “predicted mutations”) were used for further analysis. Details of mutations identified through breseq for all samples can be accessed from the website http://bugbears.ncbs.res.in/ZK819_ltsp_evol/. Mutations observed to be present at 100% frequency in all samples were considered parental mutations and were excluded from the analysis (see Table S1 in the supplemental material). The following mutants were validated by Sanger sequencing using specific primers: *cpdA* Δ3 (486 to 488), *cpdA* (L147P), *rpoS819*_92, *rpoS819*_46+92, *rpoA* (K271E), and *imp* Δ21 (Table 2). All subsequent data analyses on this set of mutations were performed using the statistical programming language R (v.3.3.0).

10.1128/mSphere.00059-17.1 TABLE S1  Preexisting mutations in the founder strain ZK819. Download TABLE S1, PDF file, 0.1 MB. Copyright © 2017 Chib et al. 2017 Chib et al. This content is distributed under the terms of the Creative Commons Attribution 4.0 International license.

### Scoring functional conservation for nonsynonymous mutations.

Sites carrying nonsynonymous mutations in protein-coding genes were tested for their level of conservation across gammaproteobacteria. Amino acids were grouped into six physicochemical classes (aliphatic, aromatic, polar, positive, negative, and special amino acids) as described earlier (45) and listed in Table S2. A mutation was classified as conservative if the substitute amino acid was in the same physicochemical class as the original. Only those mutations which were observed at a frequency higher than 5% in any sample were selected for this analysis.

10.1128/mSphere.00059-17.2 TABLE S2  Functional classification of amino acids. Download TABLE S2, PDF file, 0.04 MB. Copyright © 2017 Chib et al. 2017 Chib et al. This content is distributed under the terms of the Creative Commons Attribution 4.0 International license.

Homolog search for these proteins was carried out using *phmmer* (HMMER 3.1b1) across 246 reference proteomes listed under *Gammaproteobacteria* in UniProtKB (46, 47). The ortholog call was based on the bidirectional best-hit method using an inclusion threshold of 10^−10^ (48). A global alignment of each ortholog was performed with the query sequence using the Needleman-Wunsch algorithm implemented in EMBOSS, and orthologs that shared more than 50% identity were selected (EMBOSS:6.6.0.0) (49). A multiple sequence alignment was built using these orthologs and then used to score the conservation of each residue of the protein. MUSCLE software (v.3.8.31) was used to build the multiple sequence alignment (50). Shannon’s entropy (22), measuring the physicochemical diversity at each residue position across a protein’s orthologs, was calculated as follows:

$$H=-\overset{6}{\underset{i=1}{\sum }}f_{i}\times \log_{2}\left(f_{i}\right)$$

where *f*_*i*_ represents the frequency of each of the six categories of amino acids considered here.

This was then transformed to a conservation score using the following expression:

$$S=\frac{H_{\max}-H}{H_{\max}-H_{\min}}$$

where *H*_max_ = Shannon’s entropy of the least conserved residue of the protein and* H*_min_* =* Shannon’s entropy of the most conserved residue of the protein.

### Functional class overrepresentation test.

A functional classification of all genes in which coding region substitutions and indels were found was done using the MultiFun classification scheme (51). Six broad functional categories—namely, metabolism, information transfer, regulation, transport, cell processes, and cell structure—were considered. Only those genes listed in both MultiFun and the “Ecogene” database were considered for the analysis. This excludes 81 of the 3,398 genes functionally annotated in MultiFun. Out of the remaining 3,317, 3,041 fell in the six functional categories mentioned above and the rest were categorized as “Others.”

A statistical overrepresentation test was performed (52). The sum total of the length of all genes in a functional category was used to get the probability of a mutation occurring in any gene of that functional category in the genome. The *P* values were calculated under the binomial probability distribution for the proportion of mutations observed in genes for each functional category. The *P* values were adjusted for multiple testing using the Holm-Bonferroni method.

### Genetic diversity and models of neutral evolution.

The genetic diversity was measured in terms of Shannon’s entropy (22) of the genome. Shannon’s entropy at each substitution site of the genome was calculated as:

$$H=-\sum_{i=\left\{m,a\right\}}f_{i}\times \log_{2}\left(f_{i}\right)$$

where *f*_*i*_ represents the fraction of genomes in the population with a mutant (*m*) or the ancestral (*a*) base at the site of substitution.

The Shannon’s entropy of the genome was then calculated as:

$$G=\sum_{i=1}^{n}H_{i}$$

where *H*_*i*_ represents the Shannon’s entropy at each substitution site and *n* represents the total number of observed substitution sites in the population.

Neutral evolution simulations were performed under two models of genetic drift in a bacterial population of constant size with no recombination or *de novo* generation of novel mutations. The two models—namely, the Wright-Fisher (53, 54) and the fission (55) models—differ from one another in the rate at which the frequency of one type of individuals changes over time in the population. For both models, the change in genetic diversity over time was simulated under a two-type or a multitype system, as defined below.

In a two-type system, all observed substitutions constitute a single mutant haplotype. A haplotype is defined here as a unique copy of the bacterial chromosome that is identified by a set of SNPs in a population. The change in frequency of the mutant over a span of 20 days, if initially present at 1%, was simulated, and the fraction of simulations in which the mutant attained a frequency of 5% or above was defined as being equivalent to the *P* value for the observed genetic diversity.

In a multitype system, substitutions are distributed over several haplotypes. If the number of substitutions is much smaller than the population size, then each substitution is likely to represent one haplotype. The overall change in entropy was simulated by building an ensemble of genomes of length equal to the number of substituted sites. The simulation was begun by randomly selecting 1% of genomes to carry a substitution at each site. The ensemble was rebuilt after every generation based on the number of copies that each haplotype would leave for the next generation. Any site with a mutant frequency lower than 1% was considered to have zero entropy. To compensate for the loss of entropy from about half of the substituted sites, the *P* value was determined by the fraction of simulations in which the ensemble attained a diversity equal to or more than half of the observed genetic diversity.

Under the Wright-Fisher model, the number of descendants that each individual leaves in the next generation is approximately Poisson distributed with an average of 1 descendant/generation. With only two types of individuals, carrying either an ancestral or the mutant allele, the frequency of occurrence of each type in any given generation can be calculated from a binomial sampling of alleles from the previous generation. The maximum number of offspring left by any individual at the end of a generation is solely limited by the population size. Note that a generation here is not equivalent to the bacterial doubling time but to any span of time at the end of which the population is observed to be constant.

Under the fission model, the population size is maintained, as in any generation the number of bacteria dividing is equal to the number of bacteria perishing while the rest of the population is allowed to continue to the next generation without dividing. The birth-death probability and the number of generations govern the extent of diversity that a population attains in the given duration of time.

### Accession number(s).

Genomic deep-sequencing data can be accessed from the NCBI Sequence Read Archive under accession number SRP094816.
