# Supplementary material for: Genomewide Mutational Diversity in Escherichia coli Population Evolving in Prolonged Stationary Phase
Source: mSphere. 2017 May 24;2(3):e00059-17. doi: 10.1128/mSphere.00059-17 (PMC5444009; doi:10.1128/mSphere.00059-17)
Supplement: TABLE S1 [file sph003172291st1.pdf]

	Position	Mutation	Annotation	Gene	Function
1	66457	C→A	V32F (GTT→TTT)	araD ←	L-ribulose-5-phosphate 4-epimerase
2	97880	C→T	S265F (TCC→TTC)	murD →	UDP-N-acetylmuramoyl-L-alanine:D-glutamate ligase
3	167632	C→A	P50Q (CCG→CAG)	fhuA →	ferrichrome outer membrane transporter
4	274547	A→G	intergenic (-206/-2)	insH ← / → mmuP	IS5 transposase and trans-activator/S-methylmethionine transporter
5	274733	Δ97,240 bp		[mmuP]–[mhpD]	90 genes:[mmuP], mmuM, afuC, afuB, insB, insA, ykgN, yagB, yagA, yagE, yagF, yagG, yagH, yagI, argF, insB, insA, yagJ, yagK, yagL, yagM, yagN, intF, yagP, yagQ, yagR, yagS, yagT, yagU, ykgJ, yagV, yagW, yagX, yagY, yagZ, ykgK, ykgL, Y75_p4288, Y75_p4289, eaeH, insE, insF, ykgA, ykgB, ykgI, ykgC, ykgD, ykgE, ykgF, ykgG, ykgH, betA, betB, betI, betT, yahA, yahB, yahC, yahD, yahE, yahF, yahG, yahH, yahI, yahJ, yahK, yahL, yahM, yahN, yahO, prpR, prpB, prpC, prpD, prpE, codB, codA, cynR, cynT, cynS, cynX, lacA, lacY, lacZ, lacI, mhpR, mhpA, mhpB, mhpC, [mhpD][mmuP], mmuM, afuC, afuB, insB, insA, ykgN, yagB, yagA, yagE, yagF, yagG, yagH, yagI, argF, insB, insA, yagJ, yagK, yagL, yagM, yagN, intF, yagP, yagQ, yagR, yagS, yagT, yagU, ykgJ, yagV, yagW, yagX, yagY, yagZ, ykgK, ykgL, Y75_p4288, Y75_p4289, eaeH, insE, insF, ykgA, ykgB, ykgI, ykgC, ykgD, ykgE, ykgF, ykgG, ykgH, betA, betB, betI, betT, yahA, yahB, yahC, yahD, yahE, yahF, yahG, yahH, yahI, yahJ, yahK, yahL, yahM, yahN, yahO, prpR, prpB, prpC, prpD, prpE, codB, codA, cynR, cynT, cynS, cynX, lacA, lacY, lacZ, lacI, mhpR, mhpA, mhpB, mhpC, [mhpD]
6	547694	A→G	intergenic (+123/-1156)	fdrA → / → ylbF	acyl-CoA synthetase with NAD(P)-binding Rossmann-fold domain/hypothetical protein
7	547831	+G	intergenic (+260/-1019)	fdrA → / → ylbF	acyl-CoA synthetase with NAD(P)-binding Rossmann-fold domain/hypothetical protein
8	556858	A→T	L36Q (CTG→CAG)	folD ←	bifunctional 5,10-methylene-tetrahydrofolate dehydrogenase/5,10-methylene-tetrahydrofolate cyclohydrolase
9	569996	G→C	intergenic (+345/-120)	ybcK → / → ybcL	recombinase/kinase inhibitor
10	654214	Δ1,199 bp		insH	insH
11	749354	C→A	P17P (CCG→CCT)	ybgO ←	fimbrial-like adhesin protein
12	758440	C→A	F110L (TTC→TTA)	sdhB →	succinate dehydrogenase, FeS subunit
13	887234	C→A	A176A (GCG→GCT)	ybjJ ←	transporter
14	987574	G→T	intergenic (-170/+433)	ompF ← / ← asnS	outer membrane porin 1a (Ia;b;F)/asparaginyl tRNA synthetase
15	1093686	T→C	V130A (GTA→GCA)	ycdT →	diguanylate cyclase
16	1170573	C→T	intergenic (-164/-77)	ycfQ ← / → ycfR	DNA-binding transcriptional regulator/hypothetical protein
17	1301041	Δ1,336 bp		insC–insD	insC, insD
18	1318876	G→A	P21S (CCT→TCT)	trpA ←	tryptophan synthase subunit alpha
19	1413639	Δ23,060 bp		[ydaO]–ynaE	30 genes: [ydaO], intR, ydaQ, ydaC, lar, recT, recE, racC, ydaE, kil, sieB, ydaF, ydaG, racR, ydaS, ydaT, ydaU, ydaV, ydaW, rzpR, rzoR, trkG, ynaK, ydaY, ynaA, insH, stfR, tfaR, pinR, ynaE[ydaO], intR, ydaQ, ydaC, lar, recT, recE, racC, ydaE, kil, sieB, ydaF, ydaG, racR, ydaS, ydaT, ydaU, ydaV, ydaW, rzpR, rzoR, trkG, ynaK, ydaY, ynaA, insH, stfR, tfaR, pinR, ynaE
20	1483588	G→A	L322L (CTG→CTA)	ynbD →	phosphatase, inner membrane protein
21	1496775	Δ1 bp	coding (11/924 nt)	ydcI ←	DNA-binding transcriptional regulator
22	1615814	C→A	D202Y (GAC→TAC)	yneI ←	aldehyde dehydrogenase
23	1669599	Δ1 bp	coding (680/1221 nt)	dgsA ←	DNA-binding transcriptional repressor
24	1844073	G→A	intergenic (-224/-12)	ynjH ← / → gdhA	hypothetical protein/glutamate dehydrogenase, NADP-specific
25	1918872	T→A	S301T (TCC→ACC)	yebS →	inner membrane protein
26	1973476	G→A	L310L (CTC→CTT)	tar ←	methyl-accepting chemotaxis protein II
27	2005401	C→T	E115K (GAA→AAA)	fliC ←	flagellar filament structural protein
28	2170094	C→A	intergenic (-437/+32)	ogrK ← / ← yegR	DNA-binding transcriptional regulator/hypothetical protein
29	2320795	Δ1,336 bp		insD–insC	insD, insC
30	2406075	C→T	V51I (GTT→ATT)	nuoF ←	NADH:ubiquinone oxidoreductase, chain F
31	2416102	G→A	L233L (CTG→TTG)	yfbS ←	transporter
32	2538628	C→T	T258T (ACC→ACT)	cysK →	cysteine synthase A, O-acetylserine sulfhydrolase A subunit
33	2865274	+46 bp	coding (817/876 nt)	rpoS ←	RNA polymerase, sigma S (sigma 38) factor
34	2866111	A→G	intergenic (-21/+159)	rpoS ← / ← nlpD	RNA polymerase, sigma S (sigma 38) factor/outer membrane lipoprotein
35	3003330	G→A	L371L (CTC→CTT)	ygeV ←	DNA-binding transcriptional regulator
36	3049815	G→T	R387S (CGC→AGC)	visC ←	oxidoreductase
37	3397813	C→G	D141H (GAT→CAT)	yhdE ←	hypothetical protein
38	3742936	Δ1,336 bp		insD–insC	insD, insC
39	3746911	Δ1,199 bp		insH	insH
40	3750122	Δ1 bp	coding (365/1416 nt)	tnaA ←	tryptophanase/L-cysteine desulfhydrase, PLP-dependent
41	3898657	C→G	L259V (CTG→GTG)	yiaJ →	DNA-binding transcriptional repressor
42	3958123	Δ34 bp	coding (1156-1189/1287 r	dctA →	C4-dicarboxylic acid, orotate and citrate transporter
43	4090749	A→T	Q135L (CAG→CTG)	malQ →	4-alpha-glucanotransferase
44	4165991	A→C	K43T (AAA→ACA)	rpsL →	30S ribosomal protein S12
45	4310363	Δ1,199 bp		insH–[alsK]	insH, [alsK]
46	4371271	Δ2 bp	intergenic (-6/+299)	dcuA ← / ← aspA	C4-dicarboxylate antiporter/aspartate ammonia-lyase
47	4546535	T→A	intergenic (+296/-182)	fimB → / → fimE	tyrosine recombinase/inversion of on/off regulator of fimA/tyrosine recombinase/inversion of on/off regulator of fimA
