# Supplementary material for: Genomewide Mutational Diversity in Escherichia coli Population Evolving in Prolonged Stationary Phase
Source: mSphere. 2017 May 24;2(3):e00059-17. doi: 10.1128/mSphere.00059-17 (PMC5444009; doi:10.1128/mSphere.00059-17)
Supplement: TABLE S2 [file sph003172291st2.pdf]

**Aliphatic**	**Aromatic**	**Polar**	**Positive**	**Negative**	**Special**
Ala	Phe	Ser	Lys	Asp	Gly
Val	Tyr	Thr	Arg	Glu	Pro
Leu	Trp	Asn			
Ile	His	Gln			
Met					
Cys					
